# Supplementary material for: Content-rich biological network constructed by mining PubMed abstracts
Source: BMC Bioinformatics. 2004 Oct 8;5:147. doi: 10.1186/1471-2105-5-147 (PMC528731; doi:10.1186/1471-2105-5-147)
Supplement: Additional File 5 — The original Chilibot query results of the term "long-term potentiation (LTP)" and 22 other terms, limiting the latest references analyzed to the years 1990, 1995, 2000, and 2004. [file 1471-2105-5-147-S5.bz2 › chilibotAdditionalFile5/ltp2000/html/LTP_CAMKIV.html]

 


 **LTP** and **CAMKIV** 
  
Found 2 abstracts in PubMed,  **2 abstracts were retrieved and analyzed**.  


---

 Search Google  |
 PDF files only 
|  EDU domain only 

---

**Interactive relationship** (e.g. stimulation, inhibition, etc)

- **CaMKIV**  dependent activation of CREB is a key event in the expression of genes involved in the processes of T cell activation and neuronal long term potentiation  [ **LTP** ] .  Ref: 10191497 Biometals, 1998

- :-)

  **Parallel relationship** (e.g. studied together, co-existance, homology, etc.)

  - However, despite impaired  **LTP**  and CREB activation,  **CaMKIV**  Gr deficient mice exhibited no obvious deficits in spatial learning and memory.  Ref: 10964952 J Neurosci, 2000
